# The use of commercial computerised cognitive games in older adults: a meta-analysis

**DOI:** 10.1038/s41598-020-72281-3

**Published:** 2020-09-17

**Authors:** Bruno Bonnechère, Christelle Langley, Barbara Jacquelyn Sahakian

**Affiliations:** 1 Department of Psychiatry and Behavioural and Clinical Neurosciences, University of Cambridge, Herchel Smith Bldg, Robinson Way, Cambridge, CB2 0SZ UK; 2 Center for Research in Epidemiology, Biostatistics and Clinical Research – Public Health School, Université Libre de Bruxelles, Brussels, Belgium

**Keywords:** Cognitive ageing, Cognitive neuroscience

## Abstract

Brain training programs are currently one effective solution to prevent cognitive decline in healthy aging. We conducted a meta-analysis of randomized controlled trials assessing the use of commercially available computerised cognitive games to improve cognitive function in people aged above 60 years old without cognitive impairment. 1,543 participants from sixteen studies were included in the meta-analysis. Statistically significant improvements were observed for processing speed (SMD increased 0.40 [95% CI 0.20–0.60], p < 0.001), working memory (0.21 [95% CI 0.08–0.34], p = 0.001), executive function (0.21 [95% CI 0.06–0.35], p = 0.006), and for verbal memory (0.12 [95% CI 0.01–0.24, p = 0.031), but not for attention or visuospatial abilities. No relationship between the age of the participants and the amount of training was found. Commercially available computerised cognitive games are effective in improving cognitive function in participants without cognitive impairment aged over 60 years.

## Introduction

According to the World Health Organization (WHO), the number of people over the age of 60 years in the population will double by 2050 and is estimated to include around 2 billion people^1^. As such, the WHO has suggested that preventing cognitive decline and dementia is a global mental health priority. Dementia has a significant impact, not only on patients and relatives, but also on society. The economic cost has been estimated at €232 billion for European countries in 2015 and is expected to double by 2040)^2^.

Cognitive brain training methods have been developed for decades to preserve and increase cognitive functions of young healthy people^3^, healthy older adults^4^ and patients with Mild Cognitive Impairment (MCI)^5^. Indeed, education and life-long learning are modifiable risk factors^6,7^ and enhance cognitive reserve, which seems to provide some resilience against dementia^8,9^. A recent systematic review summarized the current level of evidence of brain training for healthy older adults and MCI. Currently, there is moderate-strength evidence for improvement of cognitive performance after cognitive training in healthy older adults, however, the transfer is low and the benefits are limited to the domain trained.

Thanks to the evolution of the technology, brain training exercises have been progressively integrated into computerized training: computers, game consoles and more recently on smartphones and tablets. One systematic review^10^ and one meta-analysis^11^ have summarized the results of studies using computerized cognitive training (CCT) or video games in healthy older adults. CCT was found to be modestly effective at improving cognitive performance in healthy older adults, though efficacy varied across cognitive domains^12^.

Recently, Cochrane’s reviews have been published, summarizing the use of CCT in various populations. In middle-aged cognitively healthy people (40–65 years old), the authors were unable to determine whether training was effective in maintaining global cognitive function^13^. In cognitively healthy people aged 65 or older, 12 or more weeks of CCT may improve cognition but the level of evidence is moderate. There are a few limitations in those studies: the first meta-analysis^12^ was published in 2014 and has not been updated since, the Cochrane’s reviews^13,14^ only included studies with training lasting for at least 12 weeks; finally, the previous reviews mixed different types of interventions (e.g., computerized training, specific games, commercial (video) games).

Recently, there has been a significant increase in the use and availability of mobile devices and apps. Amongst the most popular is mHealth apps, which are applications for mental health (29% of the mHealth apps focus on mental health diagnosis according to a WHO study)^15^. The most apparent advantages of these apps are the high availability, the level of enjoyment, usually higher compared to traditional exercises, and the possibility to monitor and visualize the evolution of the performance^16^. A systematic review synthesises the commercially available CCT solution available to prevent cognitive decline in older adults. The authors found that the study showed high methodological quality but the authors did not perform a meta-analysis^17^.

Currently, there is a lack of information about the efficacy of such kind of apps and, in more general, of commercial brain training to improve cognition. Therefore, this study aims to summarize the current level of evidence of brain training using commercial computerised cognitive games (ccCG) in healthy older adults. The second aim is to determine if there is a dose–response relationship of the training and, finally, if the age of the participants influences the outcomes.

## Results

Sixteen studies have been included in this review representing 1,543 participants (774 in the intervention group and 769 in the control group). The characteristics of the participants and interventions are presented in Table 1. The mean age of the participants is 70 (5) years old. On average the studies lasted for a median duration of 28 [P25 = 20; P75 = 40] sessions of 40 [P25 = 20; P75 = 60] min. The total duration of training is 15.3 [P25 = 9; P75 = 34] h.

**Table 1:**
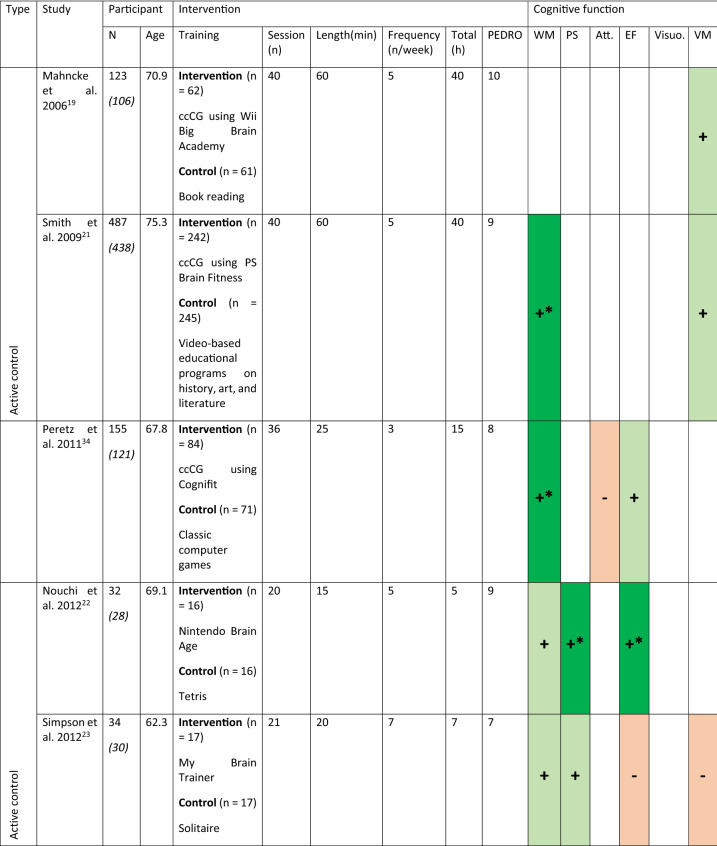

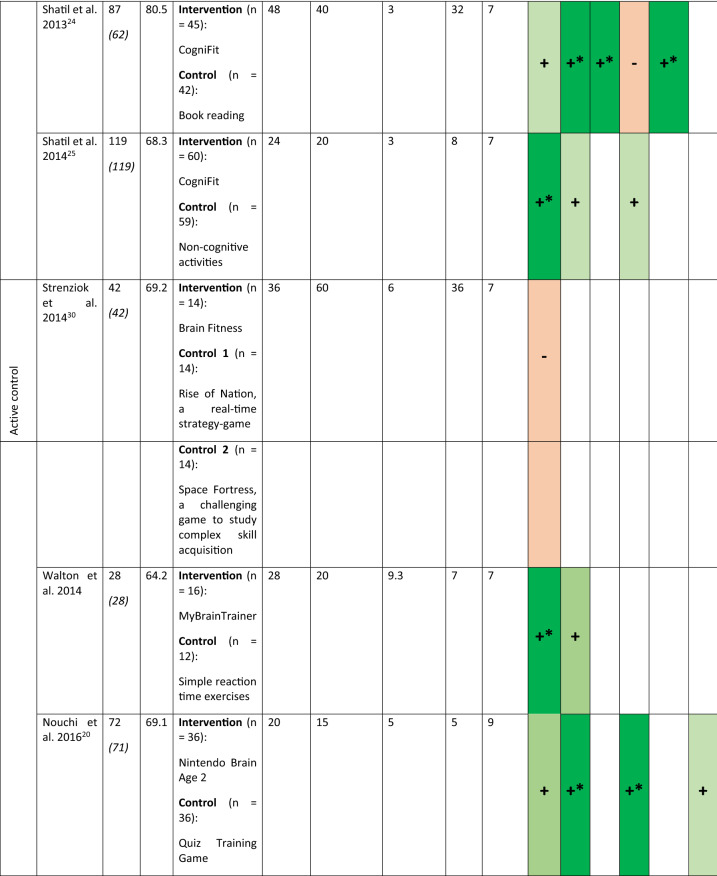

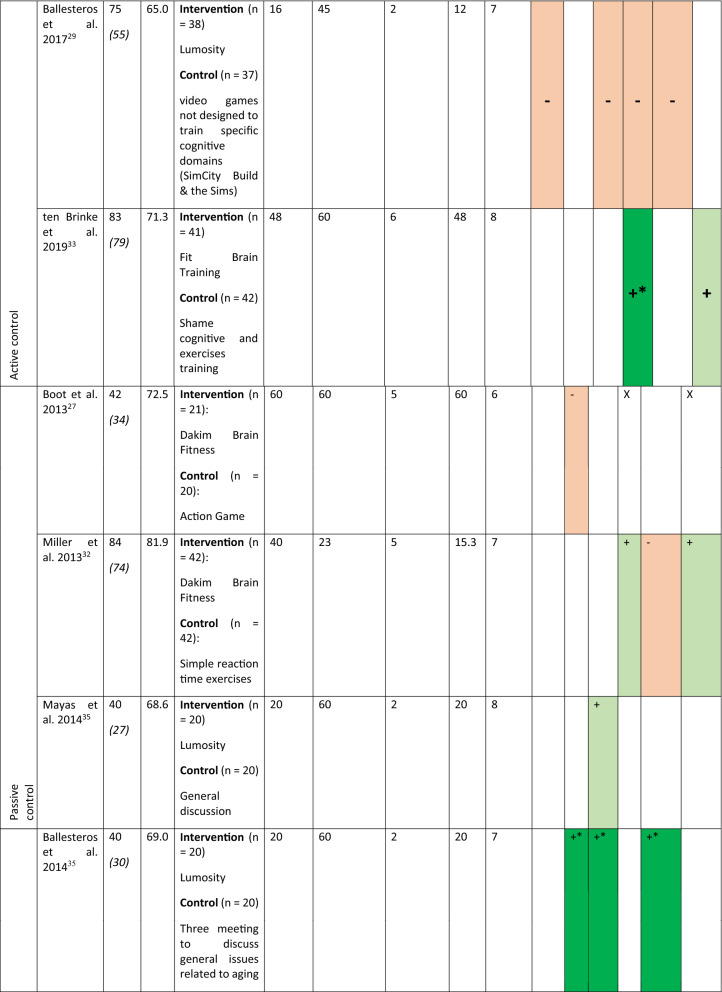
Characteristics of the participants, the interventions and the cognitive function evaluated in the included studies.

For the numbers of participants the first number indicate the participants at the inclusion, the number in italics within the parentheses is the number of participants who completed the studies.

For the cognitive assessment dark green with + * is used to indicate statistically significant results in favor of the intervention, light green with + for no-statistically significant results in favor of the intervention, dark red with -* for statistically significant results for the control group and light red with—for non-statistically significant results in favor of the control group. X indicates that the cognitive function was assessed but the SMD was equal to 0.

*WM* working memory, *PS* processing speed, *Att.* attention, *EF* executive functions, *Visuo.* visuospatial abilities, *VM* verbal memory.

**Figure 1:**
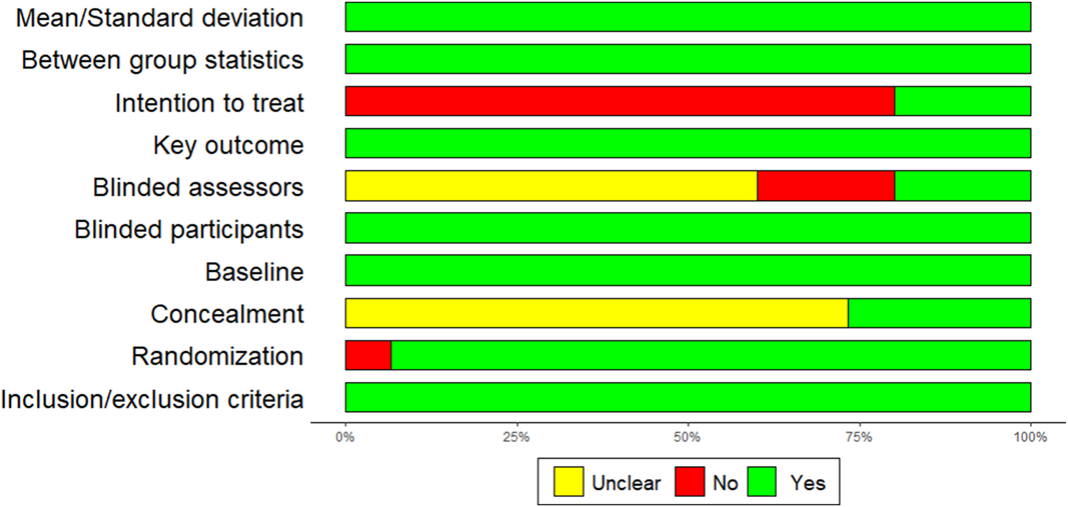
Quality of the study, author’s judgement broken down for each question of the pedros scale across all included studies.

The quality of the studies have been assessed using the PEDro scale^18^, we did not take into consideration the question about the blinding of the therapist, since the intervention are not performed with therapists, the mean score is 7.7 (1.1) out of 10. Individual results are presented in Table 1 and summarized in Fig. 1. We observed two major weaknesses in the included studies: the assessors are rarely blinded (most of the studies are single-blinded RCT) and the results of only 3 out of the 16 studies^19–21^ were analyzed in intention-to-treat. In the majority of included studies, the authors recorded drop-out at follow-up and participants did not finish the training session. Of the 1,543 participants included 1,344 (87.1%) completed the majority of the exercises and were included in the analyses (in most of the studies, the authors have defined a threshold at 80% of the amount of exercises). Of the 199 participants that did not complete the intervention 83 were in the experimental group and 116 were in the control group. There is no statistical difference in the drop-out (p = 0.41).

**Figure 2:**
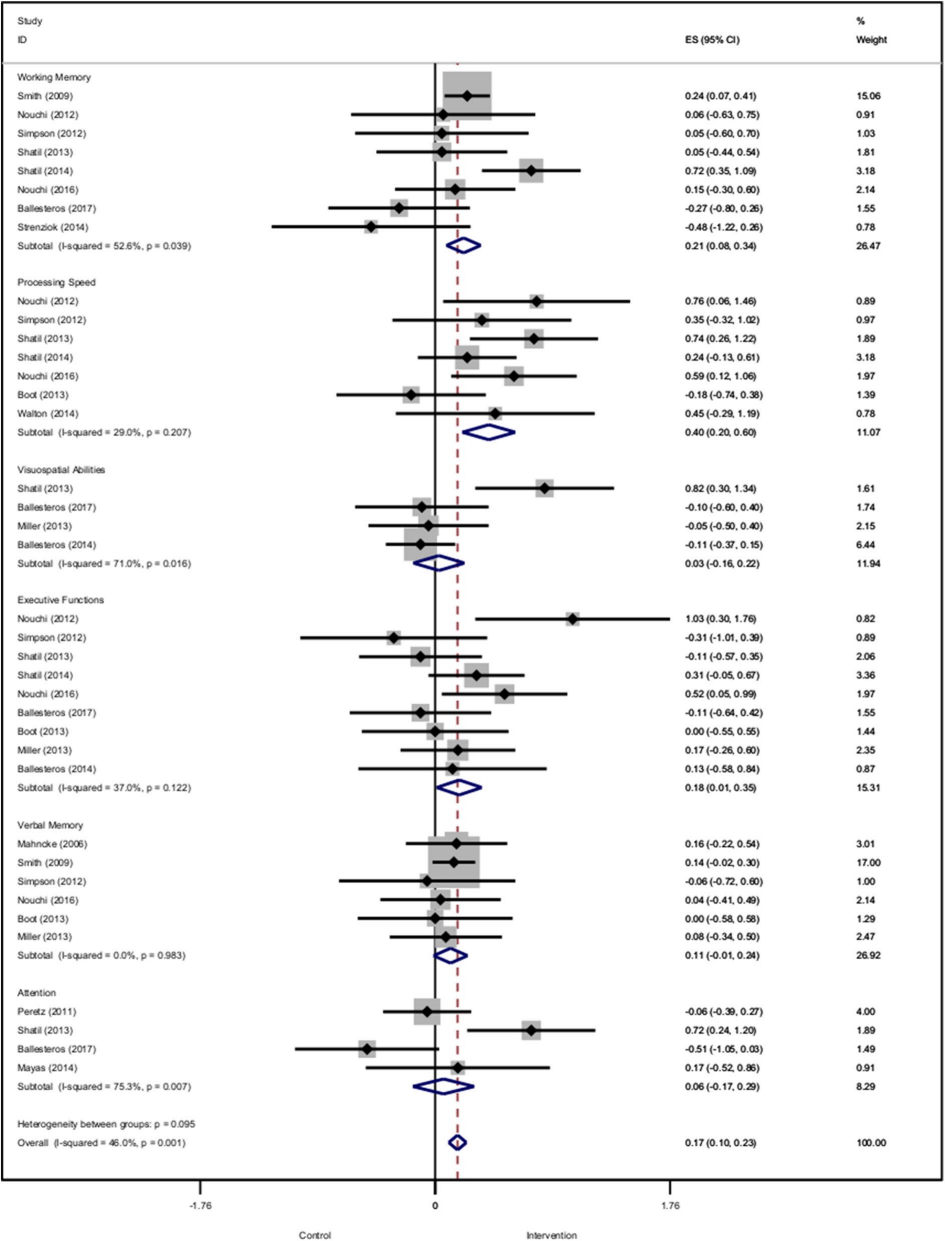
Forest plot of the included studies assessing the different cognitive functions.

Concerning the clinical efficacy, the results vary depending on the different cognitive functions evaluated. The forest plot summarizing the different studies and cognitive functions is presented in Fig. 2.

For the domain of processing speed, 8 studies, with 403 participants, were included^20,22–28^. The forest plot revealed one outlier study^28^ that reported one extremely large SMD (2.93 [2.55–3.30]). This was considered implausible (extremely large effect) and was removed from this analysis. The effect of ccCG on processing speed was moderate and statistically significant (g = 0.40 [95% CI 0.20–0.60], p < 0.001), 0.37 [0.14–0.60] after adjusting for publication bias. Heterogeneity across studies is low (tau^2^ = 0.030, p = 0.20). The funnel plot did not show significant asymmetry (Egger’s intercept = 0.67, p = 0.75).

For the domain of working memory, 9 studies, with 917 participants, were included^20–26,29,30^. The forest plot revealed two outlier studies^26,31^ that reported extremely large SMD (5.21 [3.62–6.80] and 2.93 [2.54–3.32]). This was considered implausible (extremely large effect) and was removed from this analysis. The effect of ccCG on working memory is small and statistically significant (g = 0.21 [0.08–0.34], p = 0.001), 0.28 [0.06–0.51] with the adjusted model. Heterogeneity across studies is low (tau^2^ = 0.051, p = 0.039). The funnel plot did not show significant asymmetry (Egger’s intercept = 1.02, p = 0.28).

For the domain of executive function, 9 studies, with 582 participants, were included^20,22–25,27–29,32,33^. The effect of ccCG on executive function is small and statistically significant (g = 0.21 [0.06–0.35], p = 0.006) and 0.28 [0.08–0.45] for the adjusted model. Heterogeneity across studies is low (tau^2^ = 0.040, p = 0.06). The funnel plot did not show significant asymmetry (Egger’s intercept = 1.76, p = 0.40).

For the domain of verbal memory, 7 studies, with 907 participants, were included^19–21,23,27,32,33^. The effect of ccCG on verbal memory is small and statistically significant (g = 0.12 [0.01–0.24], p = 0.031), and 0.13 [0.02–0.24] for the adjusted model. There is not heterogeneity (tau^2^ = 0, p = 0.98). The funnel plot did not show significant asymmetry (Egger’s intercept = − 0.17, p = 0.93).

For the domain of attention, 4 studies, with 299 participants, were included^24,29,34,35^. The effect of ccCG on attention is not significant (g = 0.06 [− 0.16–0.29], p = 0.59), the adjusted value is 0.12 [− 0.34–0.58]. Furthermore, the heterogeneity across studies is low (tau^2^ = 0.186, p = 0.007). The funnel plot did not show significant asymmetry (Egger’s intercept = 0.7, p = 0.90).

For the domain of visuospatial abilities, 4 studies, with 216 participants, were included^24,28,29,32^. The effect of ccCG on visuospatial abilities is not statistically significant (g = 0.03 [− 0.16–0.22], p = 0.18), the adjusted value is 0.11 [− 0.27–0.50]. Heterogeneity across studies is low (tau^2^ = 0.011, p = 0.016). The funnel plot did not show significant asymmetry (Egger’s intercept = 3.08, p = 0.42).

**Figure 3:**
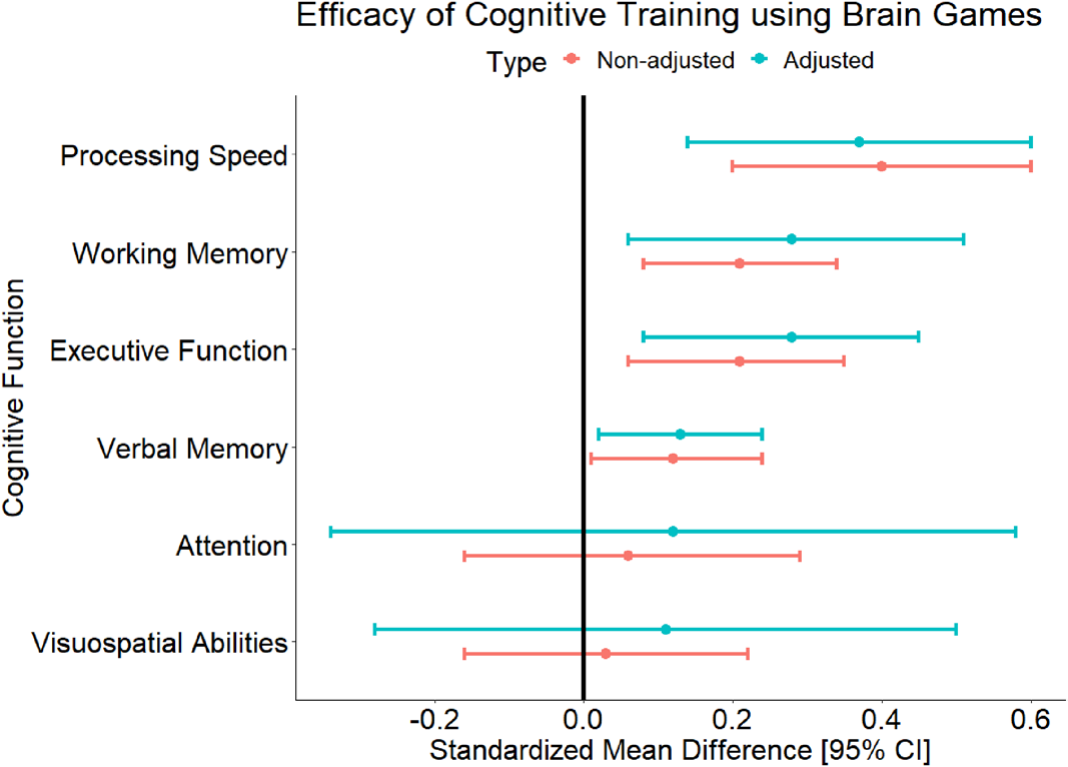
Summary of the effects size across the different cognitive functions.

The summary of the effects is presented in Fig. 3, the forest and funnel plots for the different cognitive functions are presented in Supplementary Figs. S1–S6.

**Figure 4:**
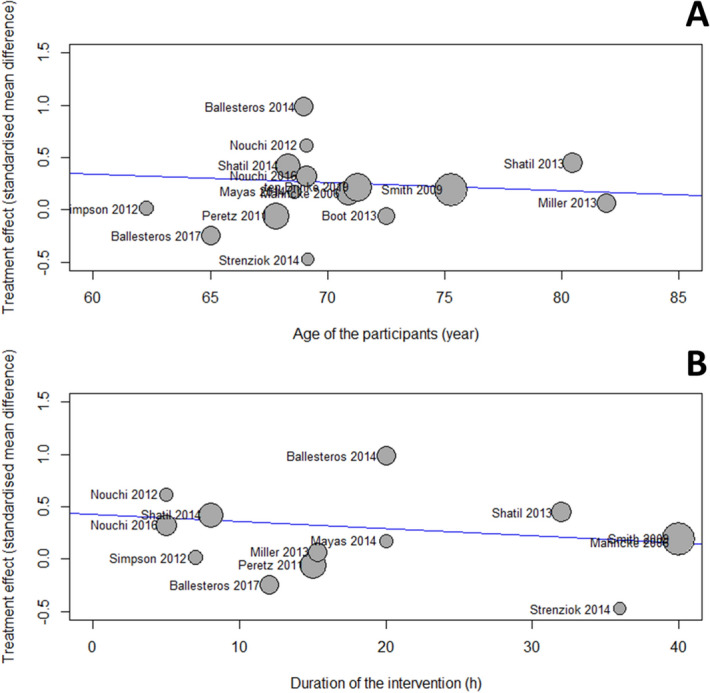
Bubble plots of the relationship between the age of the participants and the effect of the intervention (**A**), the total duration of the intervention and the effect of the intervention (**B**). Bubbles are proportional to the study weight.

We then performed meta-regression to determine if the age of the participants and the amount of training influence the outcomes. Due to the limited number of studies included (n = 16), the meta-regression analysis was conducted for the mean amongst the different cognitive functions, combined, for each study as an indicator of all cognitive outcome^12^. We did not find any significant associations between the age of the participants and the outcome (β =  − 0.008, SE = 0.020, p = 0.69) or the total duration of the training and the outcome (β =  − 0.007, SE = 0.006, p = 0.24). The results are presented in Fig. 4. The results for the different cognitive functions are presented in Supplementary Table S3 and bubble plots in Supplementary Figs. S7–S12 but are underpowered due to the small number of studies included (n < 10).

## Discussion

In this meta-analysis, despite the small numbers of studies currently published, we show that commercially available cognitive games are effective in improving processing speed, working memory, executive functions and, verbal memory of participants without cognitive impairment aged above 60 years old. However, not all cognitive functions evaluated showed improvement. In the visuospatial and attention domains, there was no statistically significant improvement compared to active or passive control interventions. It must be stressed that for these two domains, only four studies were included in each domain. Therefore the absence of results may be due to the small sample size. Our results are difficult to compare since we have highlighted the low number of studies being carried out in this field. Furthermore, only a few meta-analyses have studied the effect of CCT on different cognitive functions. In one study, authors also found a significant improvement of processing speed (SMD: 0.50 [0.14–0.87]), but unlike our study, did not find significant improvement in the executive function domain (SMD: − 0.12 [− 0.33–0.09])^11^.

There are commercial brain training games in the literature to successfully train visuospatial performance and attention (e.g., Peak Wizard and Peak Decoder)^36,37^. Visuospatial memory is a cognitive domain that is affected early in MCI and there are commercial brain training games (Peak Wizard game) shown to improve this form of memory in patient groups. Attention is increasingly being disrupted by the intrusion from technology. Gazzaley and Rosen (2016) suggest that much higher demands are now placed on our brains due to the increasingly information-saturated world (smartphones, social media, etc.) together with a growing expectation of 24/7/365 availability and immediate responsiveness^38^. The attention domain can be improved by the commercial brain training game Peak Decoder^37^. Future studies may show that these domains can be improved with these brain training games in healthy older adults people.

The biggest observed effect size, although not significantly different from the other cognitive function, was found for the processing speed. This is particularly interesting since psychomotor slowing is the cognitive domain most affected in the healthy aging process^39^, ccCG could potentially be used to slow down this decrease.

Despite the useful synthesis of the existing studies, several questions in the field of cognitive training remain. One of the most important aspects is to determine how long the benefits of this kind of intervention last. In the follow-up of the large sample size IMPACT study presented in this review^21^, the authors found that three months after completing the intervention, significant between-group differences were seen in the directly trained tasks, as well as the secondary composite outcome measures^40^. In the ACTIVE study (Advanced Cognitive Training for Independent and Vital Elderly), a large RCT on cognitive training with 2,832 participants, the authors found that compared with the control group, cognitive training resulted in improved cognitive abilities specific to the domains trained that continued 5 years after the initiation of the intervention^41^. In a 10-year follow-up study, the authors found that reasoning and speed, but not memory training, resulted in improved targeted cognitive abilities^42^. This study was partially conducted with CCT that has been now commercialized as Brain HQ. ACTIVE is one of the few cognitive training studies with older adults to employ multiple booster training sessions at 1- and 3-years post-training. The study reported significant effects from the booster training at 5 years, for reasoning and processing speed with small to moderate effects sizes (half of the magnitude of the initial training effect)^43^. For example, a single booster session counteracted 4.9 months of age-related processing speed decline^44^.

Another approach is to maintain the training over a long period. In a large scale study including 1,007 healthy older adults, the authors compared a short term intervention (32 sessions over a period of 11 weeks) and a long term intervention (192 sessions over 192 weeks)^45^. The added value of the long term intervention was that the participants had significantly improved everyday memory in contrast with the short term intensive programs whose effects decay with time. For both approaches, boosting sessions or long term training, the training could be, at least partially done, using brain games to maintain the progress obtained during conventional training sessions. Similarly to physical training the conjecture *‘use it or lose it’* is of greater importance for cognitive training and it has been highlighted that individuals who engage in training to a greater degree are more protected from relative decline^46^.

A second important question is to determine the minimum amount of training required to achieve statistical improvement. We do not find any correlation between the total amount of training and progress. Only one study assessed the relationship between the amount of training and the progress but the authors did not find a clear relationship between compliance and improvement^27^. According to this review, the median duration of the intervention is 15 h. We could thus determine this arbitrary as the ideal duration for cognitive training. It must be stressed that the duration of the training may also depend on the cognitive domain trained (e.g., processing speed to benefit sooner than executive functions), and the ideal duration and the ideal duration for a significant improvement still needs testing for the different cognitive functions.

Another important question is to identify the participants that are most likely to benefit from this kind of intervention. In the ACTIVE study, the authors found that participants with higher education and better self-rated health have greater changes in memory performance after training^47^, younger participants present more gain^48^, racial disparities in training-related gains have also been observed in this study due to variation in the external locus of control^49^. We do not have enough information about the characteristics of the participants (e.g., education level, ethnicities) in the different studies to perform a sub-group analysis and could, therefore, not confirm these findings. Besides the modifiable risk factors, genetics play an essential role in dementia and Alzheimer’s disease, in particular, the variation of APOE^50^. One study shows that the different variations of APOE also influence the effect of cognitive training in older adults without cognitive impairment^51^. It is well known that APOE ε4 is associated with an increased risk of cognitive impairment (i.e., executive function). Cognitive training may attenuate ε4-associated declines in processing speed^51^. APOE ε2 carriers, that present lower risk of cognitive impairment, may also benefit from training, particularly on measures of executive function and verbal memory, according to these authors^51^.

In addition to the issue of clinical efficacy, it is essential to ensure the safety of an intervention. Only one study reported adverse effects during brain training. In this study 487 participants trained for 40 sessions. 81 training-related adverse effects were reported (77% mild, 22% moderate, 1% severe) both physical symptoms (musculoskeletal pain, fatigue, headache) or psychological symptoms (anxiety, boredom, depressed mood), but no difference was observed between the intervention and control groups^21^. Another study mentioned that they did not record any adverse effects during the training^33^. The biggest issue with the training is that people are not performing the training. This drop-out at follow up may lead to potential bias in the studies since, in most of them, the results were not analysed in intention-to-treat. Only a few studies analysed the participants who did not complete the study and did not find differences compared with the other participants^21,24,32^. An important aspect of long-term cognitive training is keeping the participants engaged and adhering to the training over long periods. In a large study (Finnish Geriatric Intervention Study to Prevent Cognitive Impairment and Disability (FINGER)), including 631 participants with increased dementia risk, the authors indeed reported low adherence to the long term (144 sessions) CCT program. More than 200 participants did not perform any training session, 63% of the participants participated in the CCT at least once, 20% completed at least half of the training, and 12% completed all sessions^52^. This may be addressed by taking into account the level of cognitive functioning and gamification of cognitive training so that it is motivating and fun. For example, Sahakian et al.^36^ and Savulich et al.^37^, individually titrated difficulty levels and gamified episodic memory and sustained attention training and showed high levels of enjoyment and wanting to continue throughout the training.

Despite the significant results of this meta-analysis, there is a need for large sample size studies to increase the level of evidence of this kind of training. Such a large scale study has been done online with young, healthy participants (*n* = 4,715)^53^ but not yet with older adults. This raises the question as to whether this kind of training can be done in the participant’s home without supervision. Several studies have been conducted suggesting that home-based cognitive training was feasible and efficient with older adults^54–57^. Even before the development of brain training apps the efficacy of computer training and internet usage on cognitive abilities in older adults had already been highlighted^58^. Indeed compared to traditional brain training computers, smartphones, and virtual environments offer interesting possibilities indeed computerized model could bring more complex environment that will challenge and impact cognition more than traditional exercises^59^. Another potential positive aspect of having the training on smartphones is that recent studies underlined the importance of digital devices as platforms for cognitively stimulating activities in delaying cognitive decline in older adults^60^.

There are several limitations to this review. First, at the study level, some of the studies referred to have relatively small sample sizes and are likely to be underpowered^61^. It would be useful to have more large scale studies to examine the effects of cognitive training. There are other study-specific limitations (i.e., non-blinding of the assessors, the statistical analysis not performed in intention to threat, only 4 out of the 16 included studies were registered^20,22,29,35^). At the meta-analysis level, the first one is that we limited our analysis to commercially available ccCG while in the research plenty of training programs are being developed and tested but are, currently, not largely available^62–65^. We decided to include only those types of ccCG because we aimed to evaluate the efficacy of training that is available for the general public. Furthermore, in studies using commercial ccCG all the participants received the same intervention, which is not the case in studies using specific training where the treatment can be adjusted for every participant^66^. Additionally, the results from the meta-regression must be interpreted cautiously due to the small number of available studies, with more large scale studies, there may be an effect of age and the amount of training. However, the results from our meta-analysis do not provide evidence to support an effect of the age of the participants or a dose–response relationship between the amount of training and the outcome. From the clinical point of view, this is a better approach, but it is more difficult to set-up in real-life situations and in practice not always the case due to restrictions of time and financial means^67^. Again this choice has been made in the context of having the most simple solution to use, such kind of training does not require health care professionals to set-up the training and could, therefore, be of particular interest in countries with few healthcare professionals. We also limited our analysis to published papers and did not include grey literature, which could have led to an increased in the precision of the pooled estimate with narrower confidence interval^68^.

From a clinical point-of-view, we limited this analysis to purely cognitive interventions while there is a growing body of evidence suggesting than combining cognitive and physical exercises could be an effective solution to prevent cognitive decline and improve cognitive function in older adults^69–71^. A few studies also suggest that doing cognitive tasks while doing aerobic physical exercises is feasible and effective in older adults^72^.

We showed that cognitive training using commercially available ccCG is effective in improving processing speed, working memory, and executive function. The total amount of training does not seem to influence the results. In addition, the age of the participants does not influence the results, indicating that the ability to learn is preserved in healthy older adults. Only one study reported some minor adverse effects, suggesting that ccCG is safe. Therefore, in support of the findings from a previous systematic review^17^, the results of this meta-analysis support the use of ccCG to challenge the brain and improve cognitive functions. ccCG training should be combined with other methods of brain training and a healthy life-style^73^ to maintain optimal cognition and fight against the decline of cognitive functions in older adults.

Other work should focus on the use of such training to improve or slow down the cognitive decline in MCI patients as the level of evidence supporting such kind of intervention is still sparse^74,75^. There are not enough studies available to determine if ccCG can prevent clinical dementia or improve or maintain cognitive function in patients^76^.

## Methods

### Search strategy and selection criteria

We searched the PubMed electronic database, Web of Sciences, Embase, Scopus, and Sciences Direct for relevant articles published up to the 31st of December 2019. MeSH terms and free words referring to brain training (“cognitive training”, “brain training”, “memory training”, “reasoning training”, “attention training”, “processing speed training”) and games (“video games”, “exergames”, “computer training”, “games”, “mobile games”, “cognitive games”) were used as keywords. The details of the search strategies are presented in Supplementary Table S1. References from selected papers and from other relevant articles were screened for potential additional studies in accordance with the snowball principle. The search was limited to journal articles published in English.

### Eligibility criteria

The inclusion and exclusion criteria were as follows. No time period threshold was used because ccCG training is a fairly recent paradigm. A PICOS approach (Population, Intervention, Control, Outcome, and Study design) was used inclusion and exclusion criteria, which was predetermined and assessed by the study team^77^.

- *Population* Cognitively healthy participants aged above 60 years old.
- *Intervention* Studies using mobile devices or gaming consoles and using commercially available ccCG to perform cognitive training. The duration of the training must be a minimum of 1 month. Studies using action-video games or a combination of cognitive and physical rehabilitation exercises were not included.
- *Control* Active or passive brain training.
- *Outcome* Outcomes included performance on one or more cognitive tests that were not included in the training program (i.e., untrained), administered both before and after training. This review is limited to the changes in performance from baseline to immediately post-training. The primary outcomes are cognitive tests not included in the training program, administered before and after training, that provides any validated measure of on tests of verbal memory, working memory, processing speed, attention, visuospatial abilities, and executive functions. The list of the different tests used to assess the different cognitive functions in the studies is presented in Supplementary Table S2.
- *Study design* Randomized Controlled Trials.

**Figure 5:**
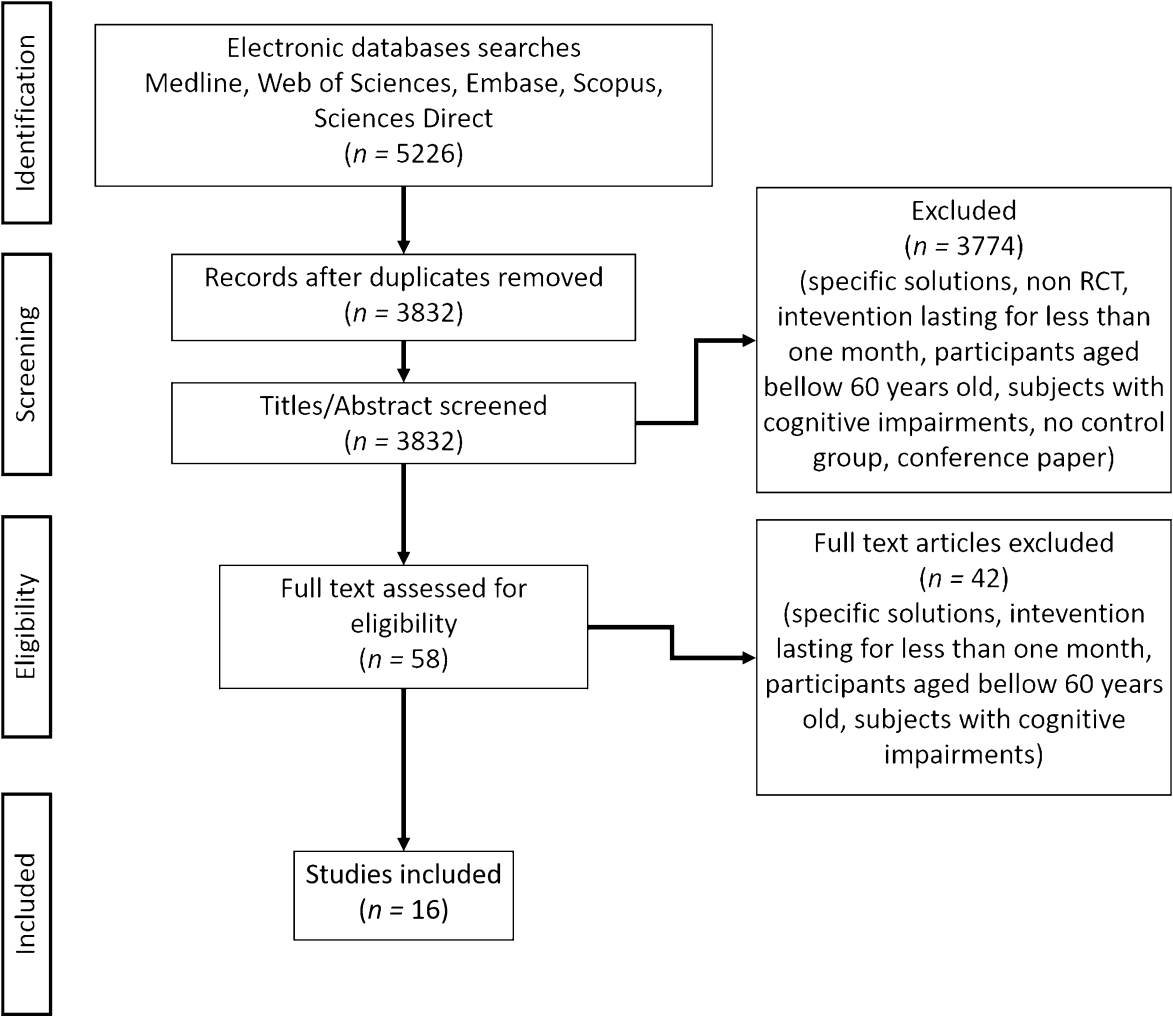
Prisma flow diagram of the studies selections.

A flow diagram of the study selection with the screened articles and the selection process is shown in Fig. 5.

### Statistical analysis

The measure of treatment effect was the effect size (standardized mean difference (SMD)), defined as the between‐group difference in mean values divided by the pooled SD computed using the Hedge’s g method. In most of the studies, several tests are used to evaluate the same cognitive function, the different results were combined together to produce a single SMD according to the Cochrane’s recommendation^78^. A positive SMD implies better therapeutic effects over time in the intervention group compared to the control group.

To detect an extreme effect size (outliers) in the different cognitive functions, two methods were used. We first checked the confidence interval of the individual studies and defined a study as an outlier if the confidence interval did not overlap with the confidence interval of the pooled effect. We then performed an influence analysis using leave-one-out method to confirm the results of the first method^79^.

We assessed the heterogeneity in stratified analyses by type of control (active or passive). We calculated the variance estimate tau^2^ as a measure of between‐trial heterogeneity^80^. We prespecified a tau^2^ of 0.0 to represent no heterogeneity, 0.0–0.2 to represent low heterogeneity, 0.2–0.4 to represent moderate heterogeneity, and above 0.4 to represent high heterogeneity between trials^81^. To assess the risk of publication bias, funnel plots were checked for asymmetry^82^ and Egger’s test for the intercept was applied for the different cognitive functions evaluated^83^. Finally, trim-and-fill method to adjust for funnel plot asymmetry and publication biases were applied^84^.

Random-effects meta-regression analysis quantified the association of the outcome and the amount of training and the age of the participants. Studies were weighted by the inverse of the sum of the within- and between-study variance^85^.

Statistical analyses were performed at an overall significance level of 0.05. Statistics were conducted in STATA (15).

### Ethical approval

The protocol of the present study was registered in the international prospective register of systematic reviews PROSPERO (registration number CRD42020167321). This systematic review and meta-analysis were reported in accordance with the Preferred Reporting Items for Systematic Reviews and Meta-Analyses (PRISMA) recommendations^86^. For the present study, no ethics committee approval was necessary.

## Supplementary information

Supplementary Information.
