# Supplementary material for: The use of commercial computerised cognitive games in older adults: a meta-analysis
Source: Sci Rep. 2020 Sep 17;10:15276. doi: 10.1038/s41598-020-72281-3 (PMC7498601; doi:10.1038/s41598-020-72281-3)
Supplement: Supplementary file 1 — Supplementary Information. [file 41598_2020_72281_MOESM1_ESM.docx]

**Supplementary Materials**

The use of commercial computerised cognitive games in older adults: a meta-analysis.

Dr. Bruno Bonnechère^1,2*^, Dr. Christelle Langley^1^, Prof. Barbara Jacquelyn Sahakian^1^

^1^ Department of Psychiatry and Behavioural and Clinical Neurosciences, University of Cambridge, Cambridge, United Kingdom CB2 0SZ

^2^ Center for Research in Epidemiology, Biostatistics and Clinical Research – Public Health School, Université Libre de Bruxelles, Brussels, Belgium


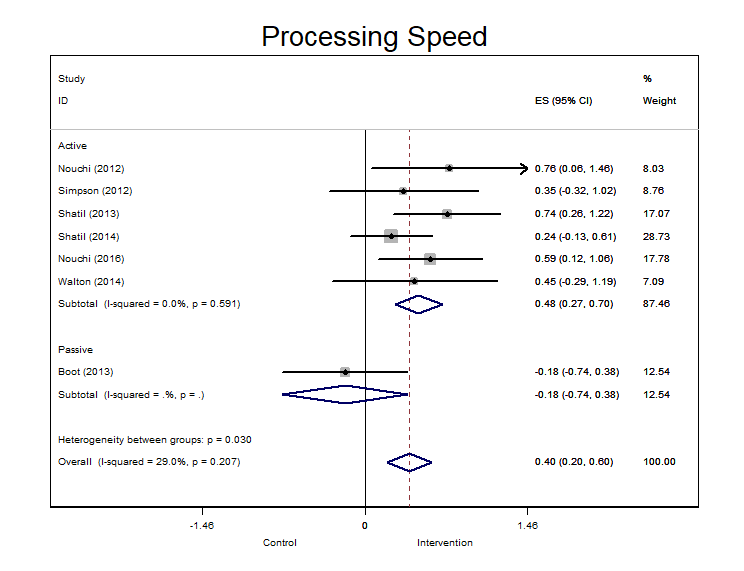

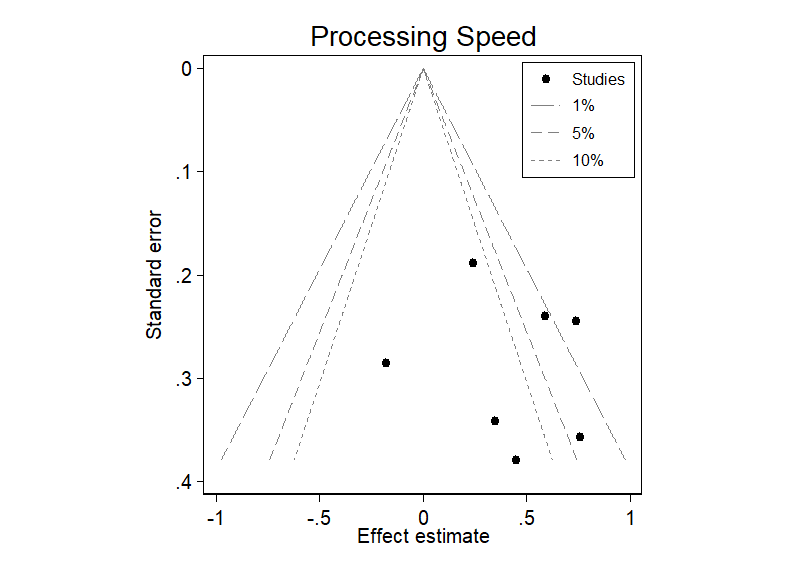


**Supplementary Figures 1: Forest and funnel plots for studies on processing speed.**


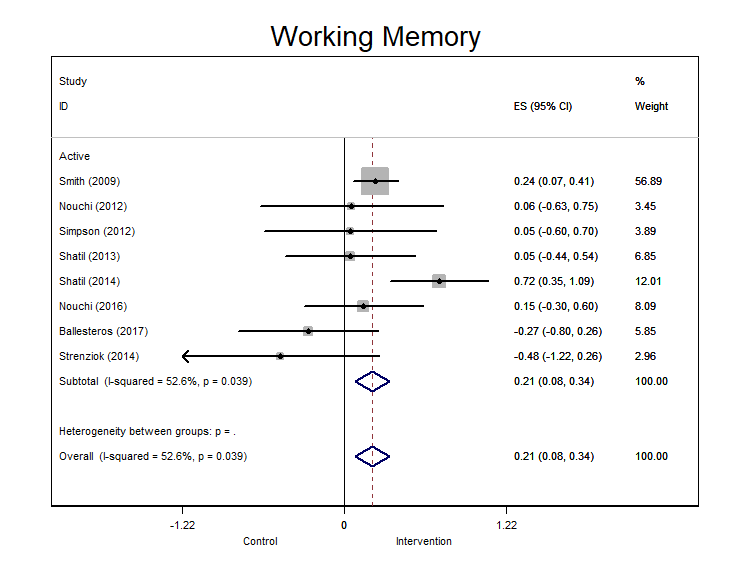

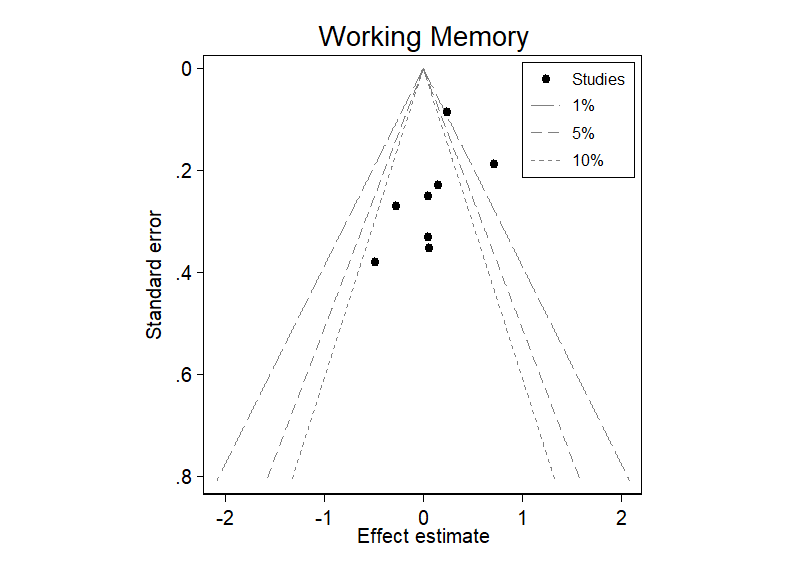


**Supplementary Figures 2: Forest and funnel plots for studies on working memory.**


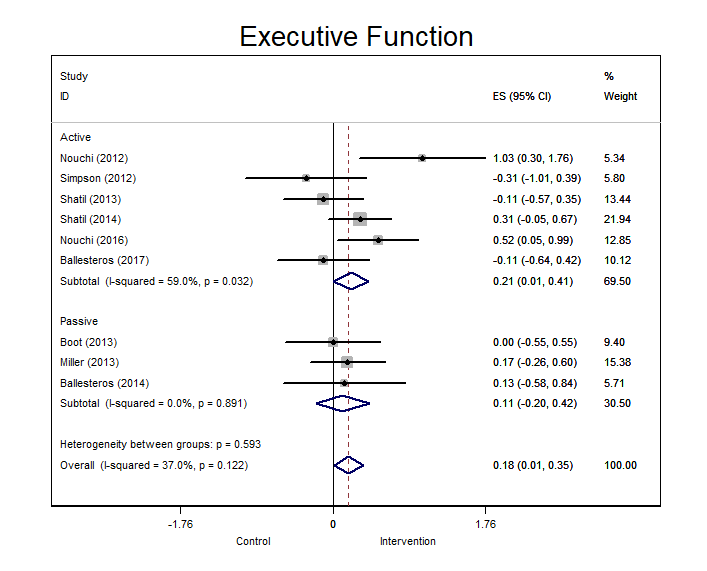

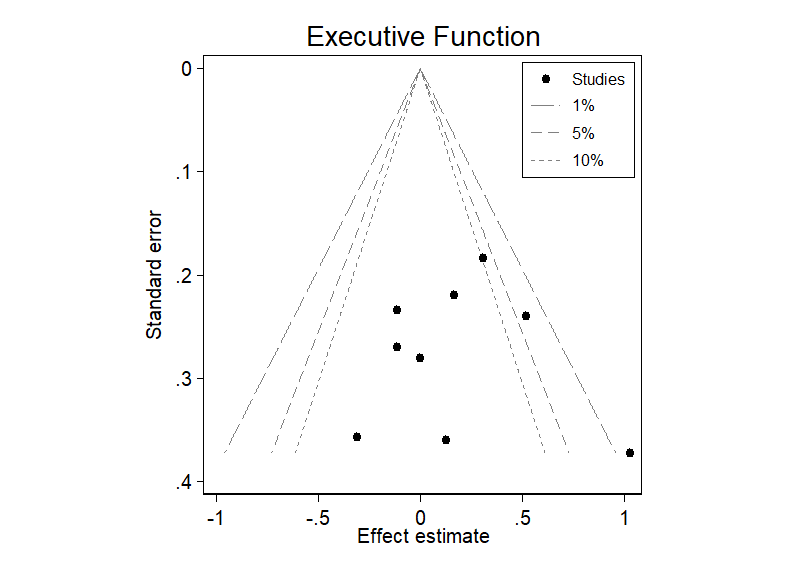


**Supplementary Figures 3: Forest and funnel plots for studies on executive function.**


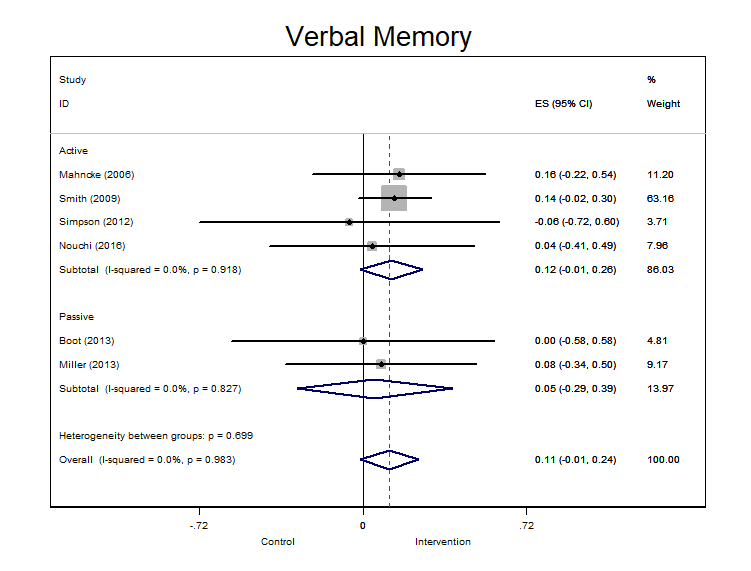

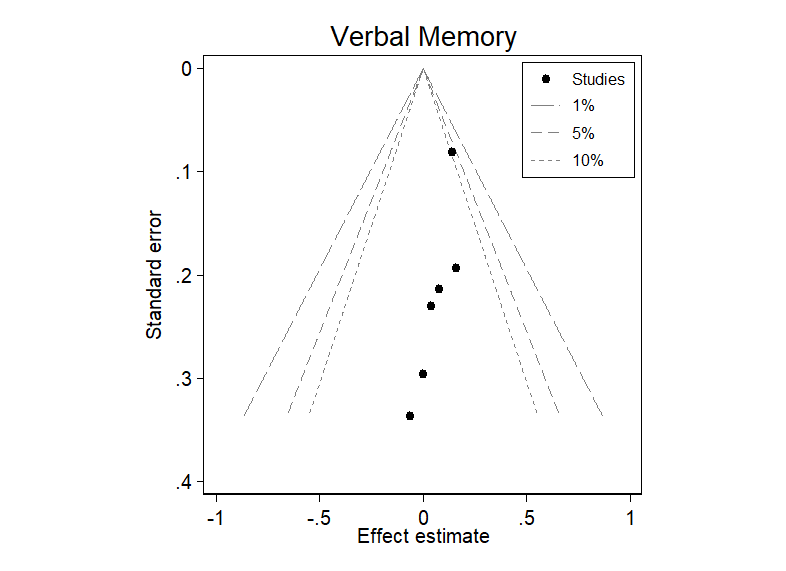


**Supplementary Figures 4: Forest and funnel plots for studies on verbal memory.**


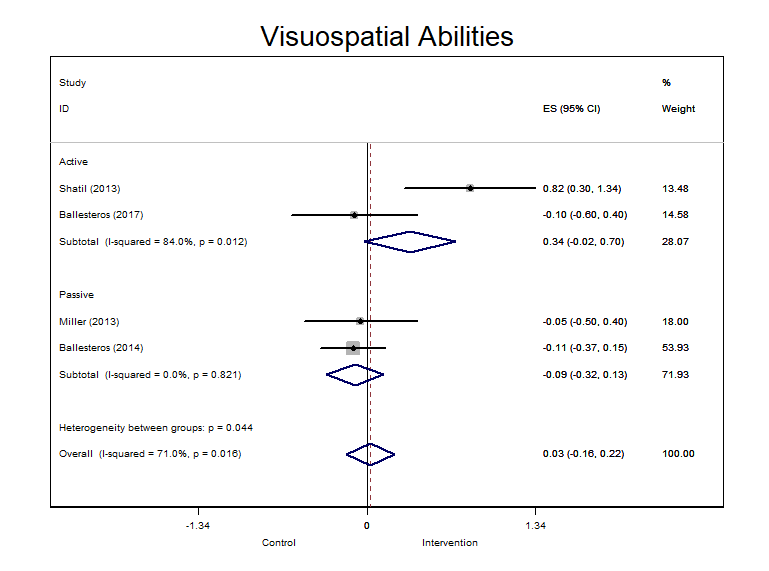

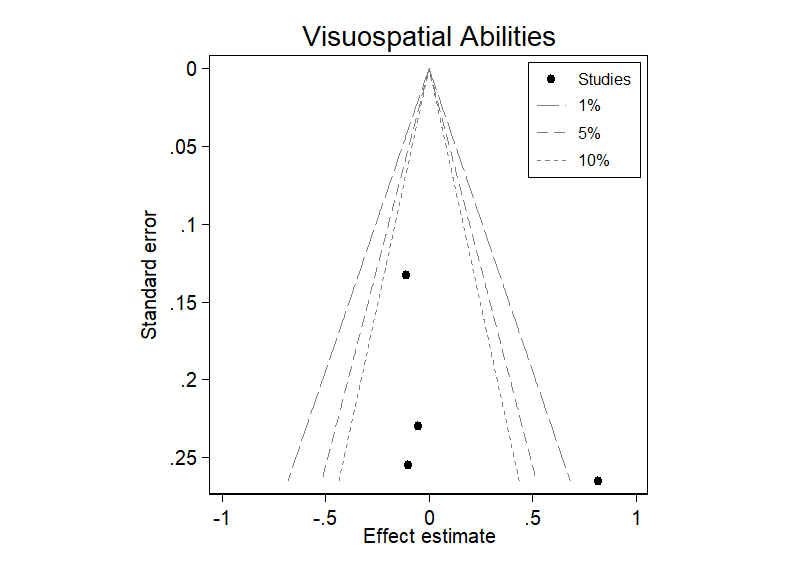


**Supplementary Figures 5: Forest and funnel plots for studies on visuospatial abilities.**


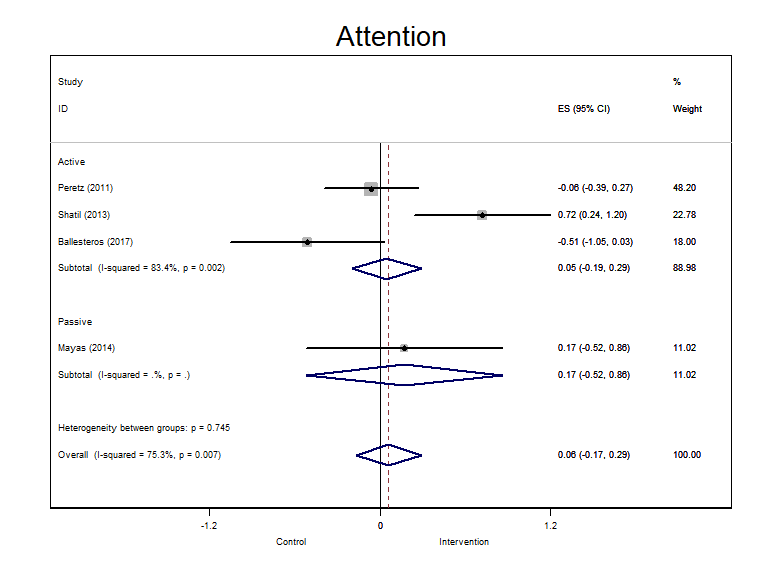

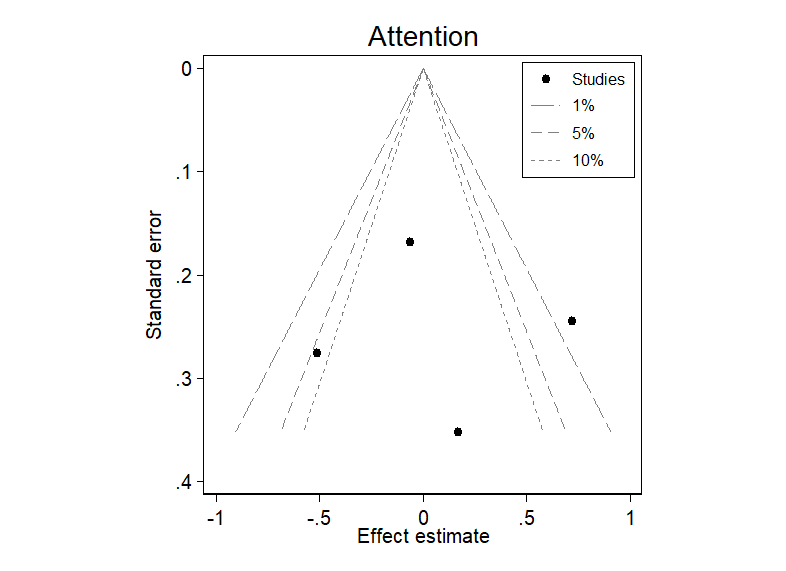


**Supplementary Figures 6: Forest and funnel plots for studies on attention.**

**
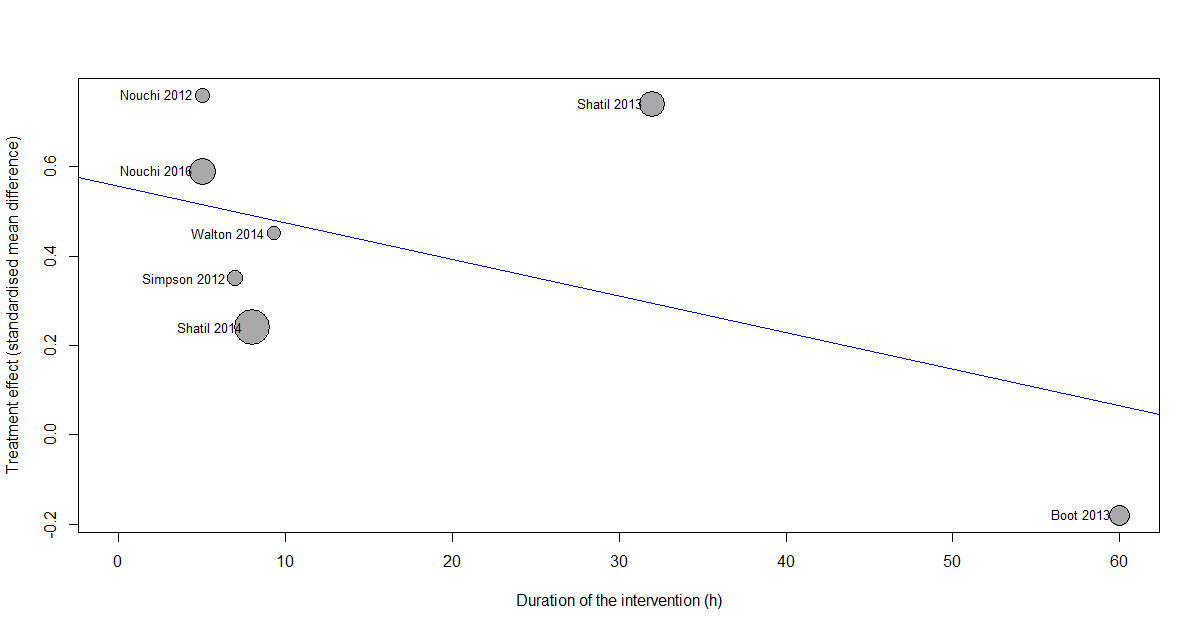
**

**
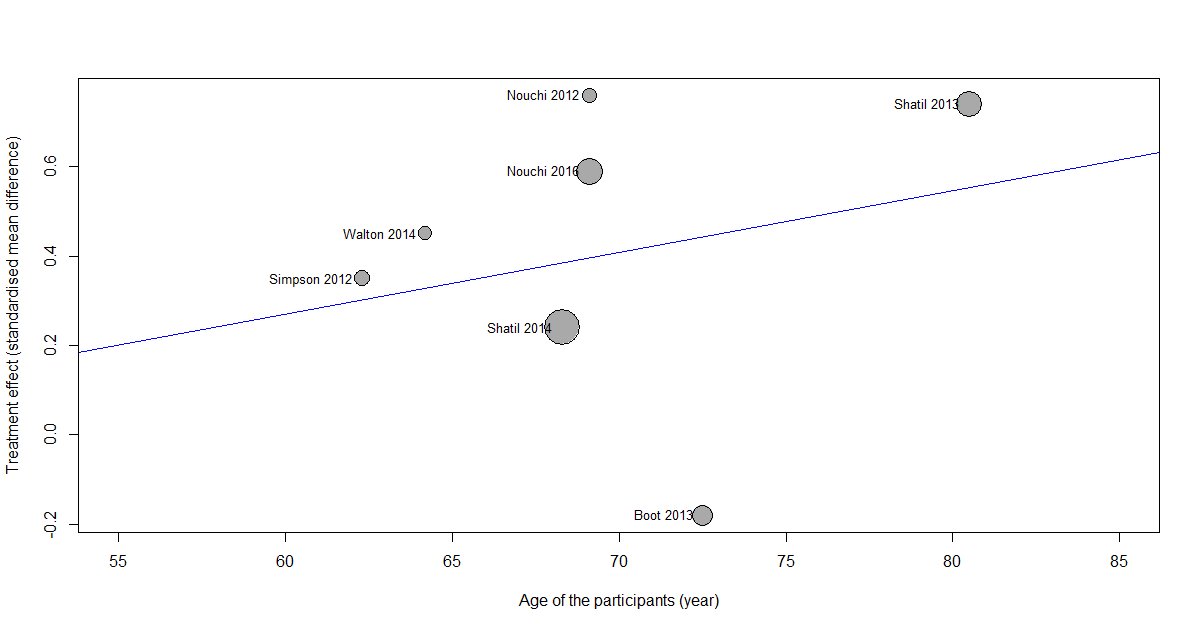
**

**Supplementary Figures 7: Association between the total duration of the training and the age of the participants and the SMD for processing speed. Bubbles are proportional to the study weight.**


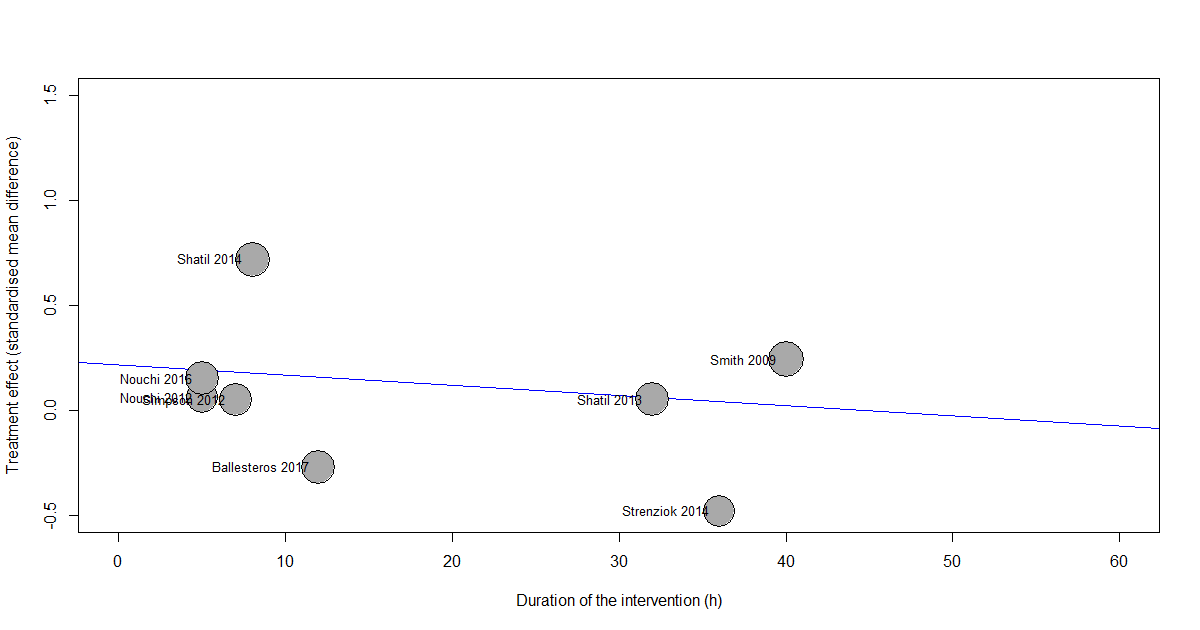


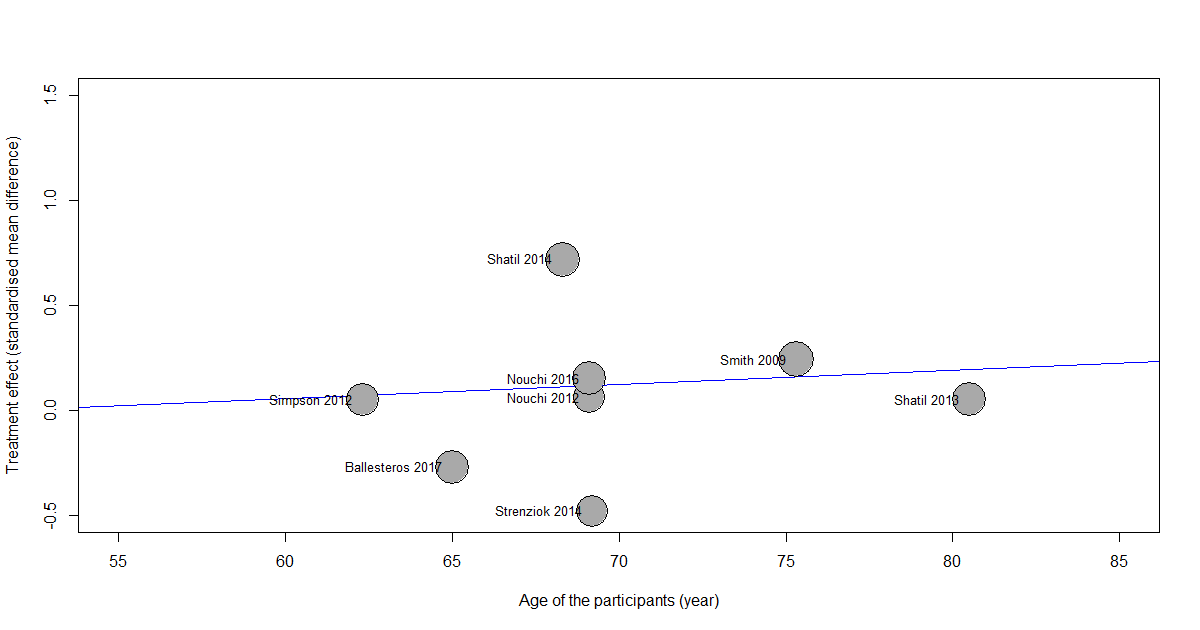


**Supplementary Figures 8: Association between the total duration of the training and the age of the participants and the SMD for working memory. Bubbles are proportional to the study weight.**

**
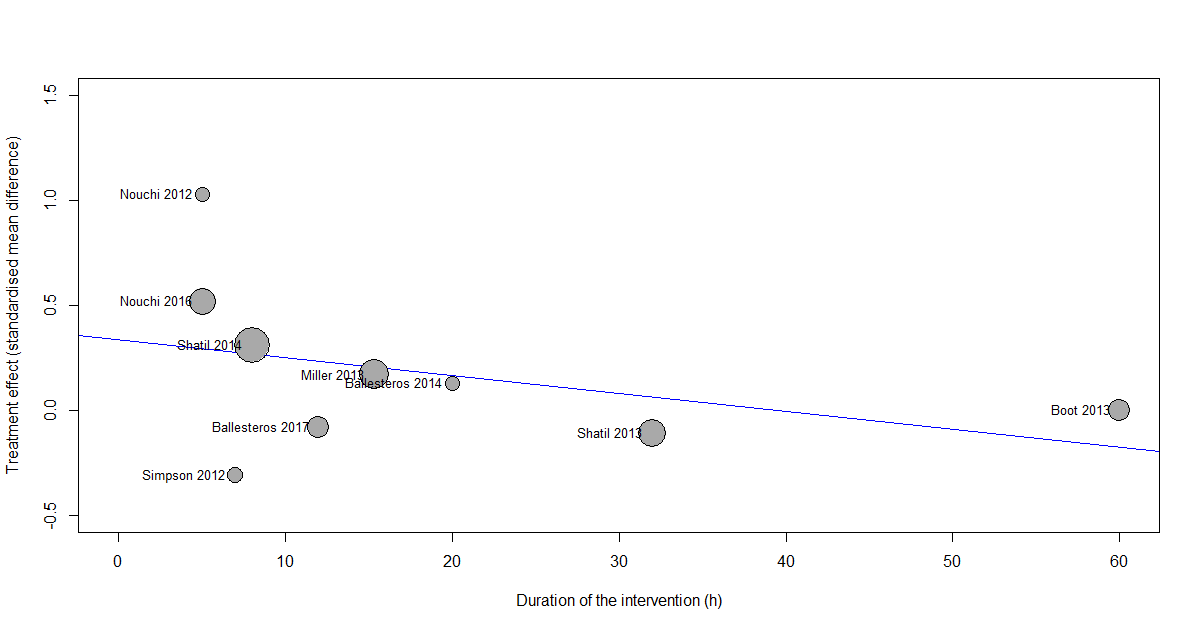
**

**
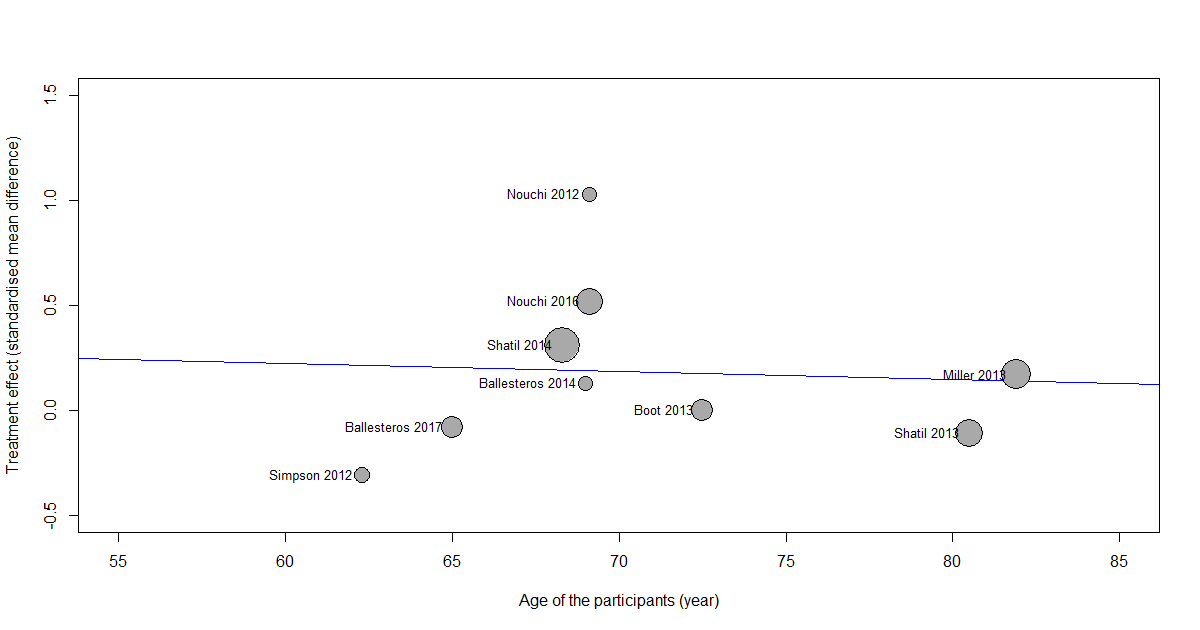
**

**Supplementary Figures 9: Association between the total duration of the training and the age of the participants and the SMD for exectuvie functions. Bubbles are proportional to the study weight.**

**
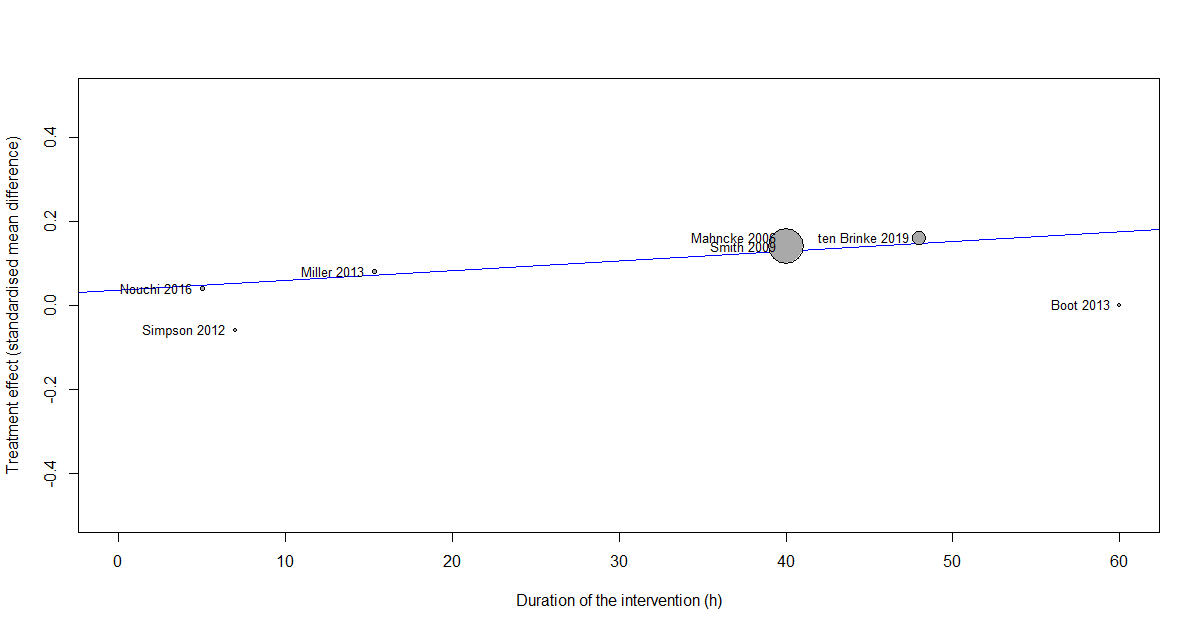
**

**
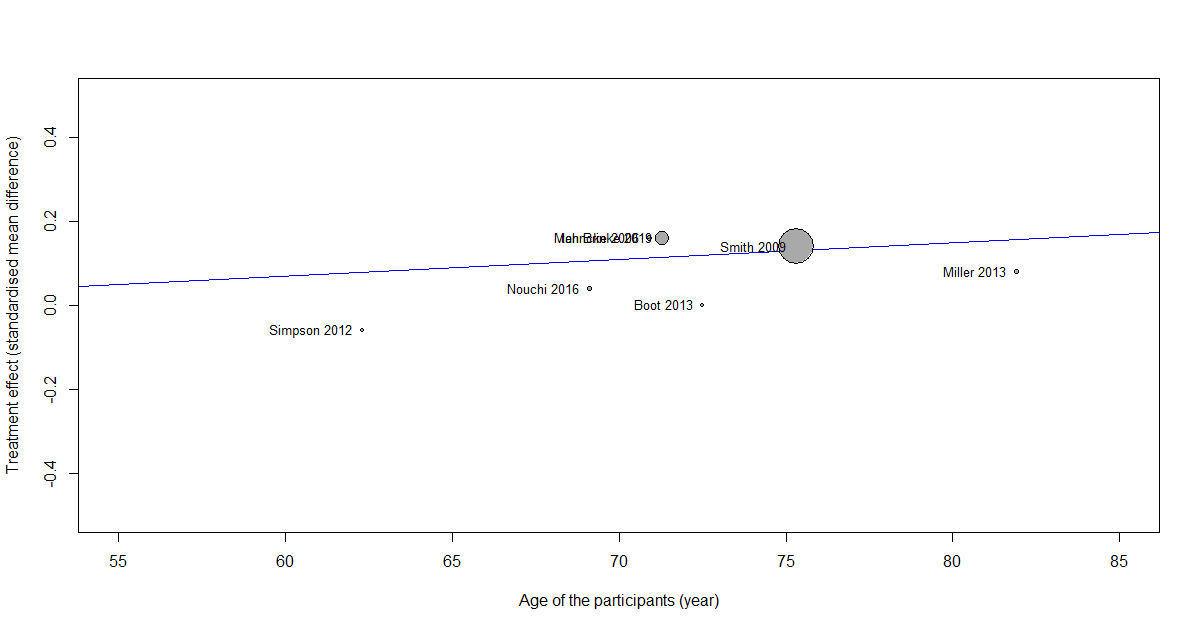
**

**Supplementary Figures 10: Association between the total duration of the training and the age of the participants and the SMD for verbal memory. Bubbles are proportional to the study weight.**

**
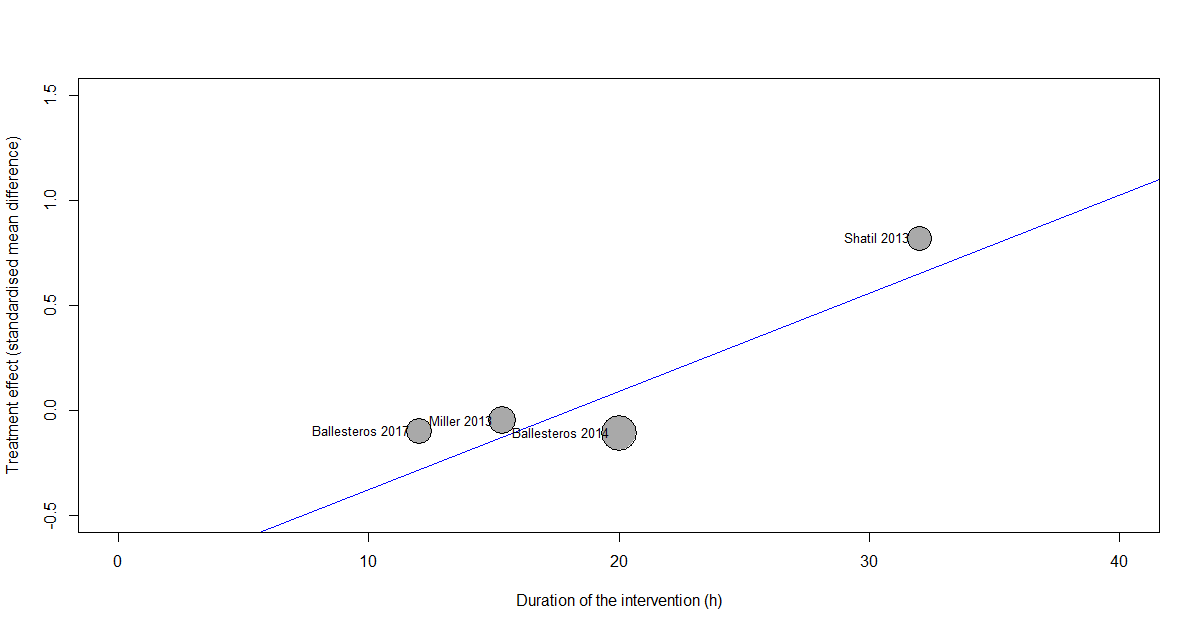
**

**
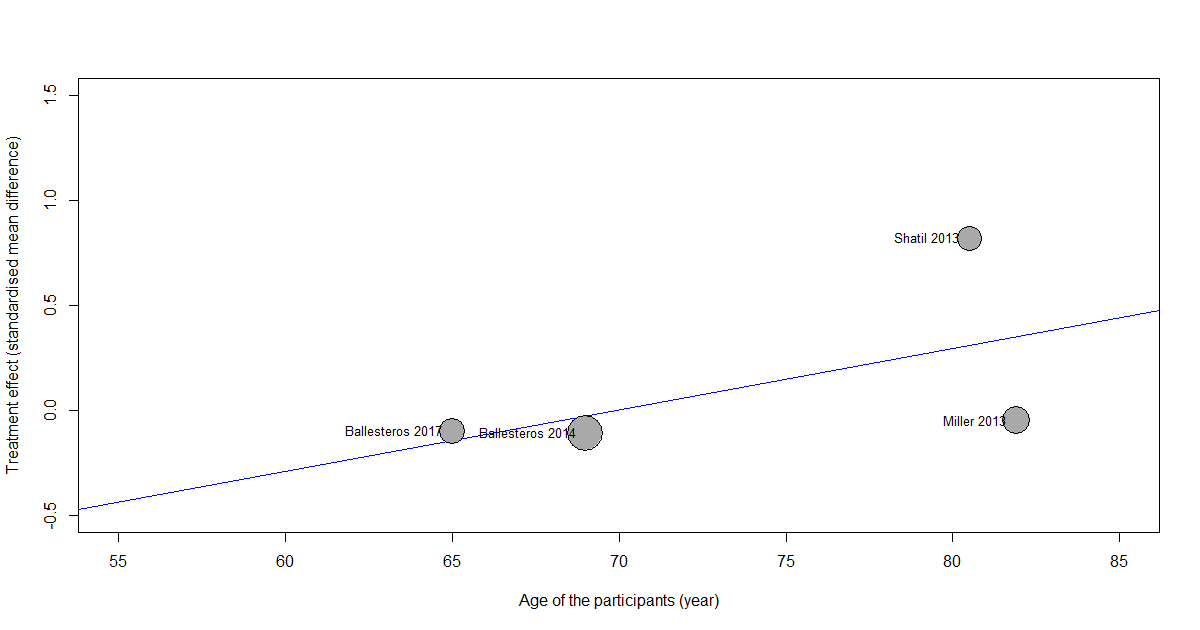
**

**Supplementary Figures 11: Association between the total duration of the training and the age of the participants and the SMD for visuospatial abilities. Bubbles are proportional to the study weight.**


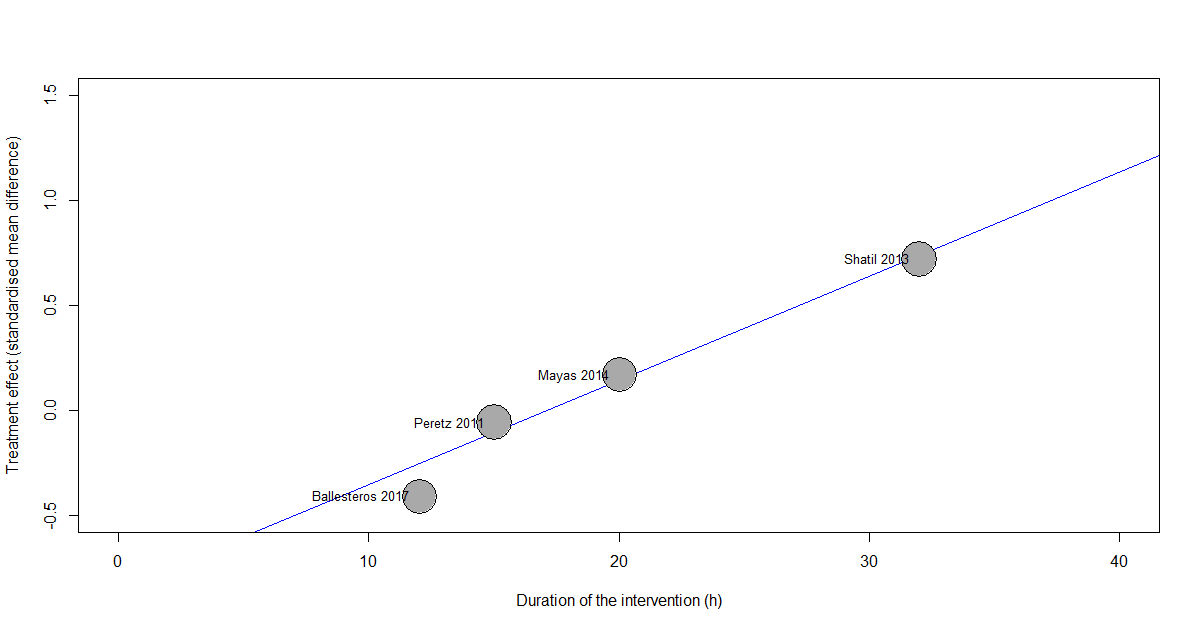

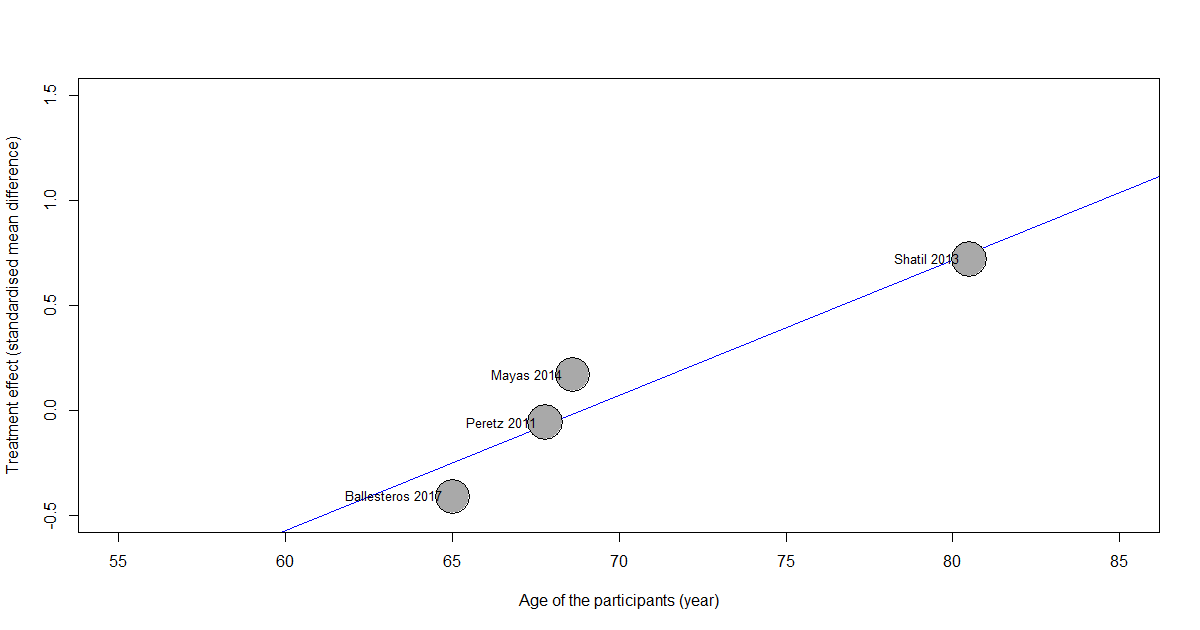


**Supplementary Figures 12:Association between the total duration of the training and the age of the participants and the SMD for visuospatial abilities. Bubbles are proportional to the study weight.**

**Supplementary Table 1: Search strategies**

| Search | Querry |
| --- | --- |
| #1 | ((((("cognition"[MeSH Terms] OR "cognition"[All Fields]) OR "cognitions"[All Fields]) OR "cognitive"[All Fields]) OR "cognitively"[All Fields]) OR "cognitives"[All Fields]) AND "education"[MeSH Terms] |
| #2 | ((("brain"[MeSH Terms] OR "brain"[All Fields]) OR "brains"[All Fields]) OR "brain s"[All Fields]) AND "education"[MeSH Terms] |
| #3 | (("learning"[MeSH Terms] OR "learning"[All Fields]) OR ("memory"[All Fields] AND "training"[All Fields])) OR "memory training"[All Fields] |
| #4 | ((((("reasoning"[All Fields]) OR "reasonings"[All Fields]) AND ((((((((("education"[MeSH Subheading] OR "education"[All Fields]) OR "training"[All Fields]) OR "education"[MeSH Terms]) OR "train"[All Fields]) OR "train s"[All Fields]) OR "trained"[All Fields]) OR "training s"[All Fields]) OR "trainings"[All Fields]) OR "trains"[All Fields]) |
| #5 | ((((((("attention"[MeSH Terms] OR "attention"[All Fields]) OR "attentions"[All Fields]) OR "attention s"[All Fields]) OR "attentional"[All Fields]) OR "attentive"[All Fields]) OR "attentively"[All Fields]) OR "attentiveness"[All Fields]) AND ((((((((("education"[MeSH Subheading] OR "education"[All Fields]) OR "training"[All Fields]) OR "education"[MeSH Terms]) OR "train"[All Fields]) OR "train s"[All Fields]) OR "trained"[All Fields]) OR "training s"[All Fields]) OR "trainings"[All Fields]) OR "trains"[All Fields]) |
| #6 | ((((("processing"[All Fields]) OR "processings"[All Fields]) AND ((("speed"[All Fields] OR "speeded"[All Fields]) OR "speeding"[All Fields]) OR "speeds"[All Fields]) AND ((((((((("education"[MeSH Subheading] OR "education"[All Fields]) OR "training"[All Fields]) OR "education"[MeSH Terms]) OR "train"[All Fields]) OR "train s"[All Fields]) OR "trained"[All Fields]) OR "training s"[All Fields]) OR "trainings"[All Fields]) OR "trains"[All Fields]) |
| #7 | ("video games"[MeSH Terms] OR ("video"[All Fields] AND "games"[All Fields])) OR "video games"[All Fields] |
| #8 | "exergame"[All Fields] OR "exergamers"[All Fields] OR "exergames"[All Fields] OR "exergaming"[All Fields] |
| #9 | (((((((((("computer s"[All Fields]) OR "computers"[MeSH Terms]) OR "computers"[All Fields]) OR "computer"[All Fields]) OR "computes"[All Fields]) OR "computing"[All Fields]) OR "computional"[All Fields]) AND ((((((((("education"[MeSH Subheading] OR "education"[All Fields]) OR "training"[All Fields]) OR "education"[MeSH Terms]) OR "train"[All Fields]) OR "train s"[All Fields]) OR "trained"[All Fields]) OR "training s"[All Fields]) OR "trainings"[All Fields]) OR "trains"[All Fields]) |
| #10 | "game s"[All Fields] OR "games"[All Fields] OR "gaming"[All Fields] |
| #11 | ("mobile"[All Fields] OR "mobiles"[All Fields]) AND (("game s"[All Fields] OR "games"[All Fields]) OR "gaming"[All Fields]) |
| #12 | ((((("cognition"[MeSH Terms] OR "cognition"[All Fields]) OR "cognitions"[All Fields]) OR "cognitive"[All Fields]) OR "cognitively"[All Fields]) OR "cognitives"[All Fields]) AND (("game s"[All Fields] OR "games"[All Fields]) OR "gaming"[All Fields]) |
| #13 | #1 OR #2 OR #3 OR#4 OR#5 OR #6 |
| #14 | #7 OR #8 OR #9 OR#10 OR#11 OR #12 |
| #15 | "Randomized Controlled Trial" [Publication Type] OR "Randomized Controlled Trials as Topic"[Mesh] |
| #16 | Random* |
| #17 | #15 OR #16 |
| #18 | #13 AND #14 AND #17 |

**Supplementary Table 2: Summary of the tests used to report the different domains of the cognitive functions.**

| Study | Cognitive function | | | | | |
| --- | --- | --- | --- | --- | --- | --- |
|  | WM | PS | Att. | EF | Visuo. | VM |
| Mahncke et al. 2006 |  |  |  |  |  | Subset of the Repeatable Battery for the Assessment of Neuropsychological Status (RBANS) |
| Smith et al. 2009 | Rivermead Behavioral Memory Test  (RBMT) immediate and delayed recall, and Wechsler Memory Scale (WMS-III) letter number  sequencing (LNS) and digit span backwards tests. An overall composite score  (Overall Memory) combining RAVLT total score and word list delayed recall, RBMT  immediate and delayed recall, and LNS and digits backwards was derived as described for  RBANS Auditory Memory/Attention |  |  |  |  | Repeatable Battery for the Assessment of Neuropsychological Status (RBANS). Rey Auditory Verbal Learning Test (RAVLT) |
| Peretz et al. 2011 | Neuropsychological Examination – CogniFit Personal  Coach previously validated to assess different cognitive functions |  | Neuropsychological Examination – CogniFit Personal  Coach previously validated to assess different cognitive functions | Neuropsychological Examination – CogniFit Personal  Coach previously validated to assess different cognitive functions |  |  |
| Nouchi et al. 2012 | Digit Cancellation  Task (D-CAT), Digit Span Forward (DS-F) Subtests of WAIS III | Digit Symbol Coding and Symbol Search |  | Frontal  Assessment Battery at bedside (FAB) and Trail Making Test-  B (TMT-B) |  |  |
| Simpson et al. 2012 | the Swinburne University Computerised Cognitive Ageing Battery (SUCCAB) computerized version  of the WM task, participants were required to remember spatial locations on a four-by-four  grid.  The Digit Symbol-Substitution test (WAIS II) | SUCCAB Simple reaction time  A single white square was presented in the  middle of the computer screen.  Complex Reaction Time:  Either a red square or a blue triangle appeared in  the centre of the computer screen. Participants  were required to press the right button (red) as  quickly as possible upon presentation of a red  square, or the left button (blue) as quickly as  possible upon presentation of the blue triangle |  | Trail making test (TMT-A & TMT-B) |  | Digit span forward and backward. (WAIS-III) |
| Shatil et al. 2013 | The CogniFit neuropsychological evaluation, requiring three 15-min sessions to administer has been used. It is composed of 15 evaluation tasks measuring a wide range of cognitive abilities such as focused and divided attention, inhibition, shifting, planning, working memory, and eye-hand coordination. Scores are derived from response times (in milliseconds) and accuracy (%). | | | | |  |
| Shatil et al. 2014 | Digit span forward and reverse version Subtests of WAIS III | Digit span forward and reverse version |  | Trail making test (TMT-A & TMT-B) |  |  |
| Strenziok et al. 2014 | Spatial working memory was assessed with an information processing  visuospatial delayed match-to-sample task. Auditory working  memory was assessed by the Letter Number Sequencing subtest of  the Wechsler Adult Intelligence Scale III (WAIS III) |  |  |  |  |  |
| Walton et al. 2014 | SUCCAB  Spatial Working Memory:  of the WM task, participants were required to remember spatial locations on a four-by-four  grid.  Contextual Working Memory:  A series of twenty everyday images (e.g., food,  tools) were presented at the top, bottom, left  or right of the screen, for three seconds each.  On completion of the series of images, they  were presented again in a randomized order  in the centre of the screen for two seconds  each. Participants were required to respond by  pressing top, bottom, left or right depending  on where the images were originally presented. | SUCCAB Simple reaction time  A single white square was presented in the  middle of the computer screen.  Complex Reaction Time:  Either a red square or a blue triangle appeared in  the centre of the computer screen. Participants  were required to press the right button (red) as  quickly as possible upon presentation of a red  square, or the left button (blue) as quickly as  possible upon presentation of the blue triangle |  |  |  |  |
| Nouchi et al. 2016 | Digit span backward (DS-B). Subtests of WAIS III | Digit symbol coding and symbol search from WAIS-III |  | To measure shifting ability in executive function, the letter fluency task (LFT) and category fluency task were used. To measure inhibition ability in executive function, Stroop task and reverse Stroop task were used. |  | Digit span forward (DS-F), subset of WAIS III |
| Ballesteros et al. 2017 | Corsi blocks task |  | Cross-modal oddball attention task | Stroop-negative priming task | N-back memory task |  |
| ten Brinke et al. 2019 |  |  |  | Stroop Colour-Word Test  Trail Making Test (Parts A&B) |  | Rey Auditory Verbal  Learning Test (RAVLT) |
| Boot et al. 2013 |  | Simple and choice reaction time Participants saw a square appear at the center of the screen and were asked to respond quickly when they saw it (simpleRT), or pushed one of two keys depending on which side of the screen the square appeared on (choiceRT)  Number comparison. Participants had to judge as quickly as possible whether the two strings of numbers were the same or different. |  |  |  | Everyday recognition  Participants were given stimuli such as banking statements and prescription labels to remember. They had 1 min to memorize these materials, and 1 min to answer questions about the memorized materials. |
| Miller et al. 2013 |  |  |  | Buschke-Fuld Selective  Reminding Test | Rey-Osterrieth Complex  Figure Test | Verbal Pairs Subtest from the WAI-III |
| Mayas et al. 2014 |  |  | Oddball task:  participants categorized a visual digit (1–8) as odd or even by  pressing response keys (V & B, counterbalanced across participants)  with two fingers from their dominant hand |  |  |  |
| Ballesteros et al. 2014 |  | Simple and choice reaction time | Cross-modal oddball attention task |  | Jigsaw-puzzle task Rey-Osterrieth complex figure test |  |

WM: Working Memory, PS: Processing Speed, Att.: Attention, EF: Executive Functions, Visuo.: Visuospatial Abilities, VM: Verbal Memory

Supplementary Table 3: Meta-Regressions between the effect of the training, the age and the total duration of the intervention

| Cognitive function | Age | | Total duration (h) | |
| --- | --- | --- | --- | --- |
|  | Β [95% CI] | p-value | Β [95% CI] | p-value |
| Processing Speed (*n =* 7) | 0.014  [-0.033 – 0.061] | 0.56 | -0.008  [-0.020 – 0.004] | 0.19 |
| Working Memory (*n =* 8) | 0.007  [-0.042 – 0.052] | 0.78 | -0.005  [-0.023 – 0.014] | 0.61 |
| Executive Function (*n =* 9) | -0.004  [-0.041 – 0.032] | 0.83 | -0.008  [-0.021 – 0.004] | 0.18 |
| Verbal Memory (*n =* 7) | 0.004  [-0.027 – 0.035] | 0.80 | 0.002  [-0.006 – 0.011] | 0.59 |
| Visuospatial Abilities (n = 4) | 0.029  [-0.026 – 0.084] | 0.30 | 0.046  [0.008 – 0.083] | 0.017 |
| Attention (*n =* 4) | 0.064  [0.029 – 0.106] | 0.002 | 0.049  [0.018 – 0.081] | 0.002 |

Note that the minimal number of studies required to perform reliable meta-regression analysis is 8-10. Therefore those results must be interpreted carefully and are presented for information, especially for visuospatial abilities and attention.
